# The Rendezvous Technique: A Minimally Invasive Non-Surgical Approach for the Management of Iatrogenic Ureteral Injuries

**DOI:** 10.3390/jcm13133820

**Published:** 2024-06-28

**Authors:** Eliodoro Faiella, Giuseppina Pacella, Elva Vergantino, Domiziana Santucci, Carlo De Cicco Nardone, Corrado Terranova, Francesco Plotti, Roberto Angioli, Bruno Beomonte Zobel, Rosario Francesco Grasso

**Affiliations:** 1 Operative Research Unit of Radiology and Interventional Radiology, Fondazione Policlinico Universitario Campus Bio-Medico, Via Alvaro del Portillo 200, 00128 Rome, Italy; g.pacella@policlinicocampus.it (G.P.); elva.vergantino@unicampus.it (E.V.); d.santucci@policlinicocampus.it (D.S.); b.zobel@policlinicocampus.it (B.B.Z.); r.grasso@policlinicocampus.it (R.F.G.); 2 Research Unit of Radiology and Interventional Radiology, Department of Medicine and Surgery, Università Campus Bio-Medico di Roma, Via Alvaro del Portillo 21, 00128 Rome, Italy; 3 Department of Obstetrics and Gynaecology, Fondazione Policlinico Universitario Campus Bio-Medico, Via Alvaro del Portillo 200, 00128 Rome, Italy; c.decicconardone@policlinicocampus.it (C.D.C.N.); c.terranova@policlinicocampus.it (C.T.); f.plotti@policlinicocampus.it (F.P.); r.angioli@policlinicocampus.it (R.A.)

**Keywords:** iatrogenic ureteral injuries, CT urography, antegrade pyelogram, double J stent, rendezvous technique

## Abstract

**Background/Objectives**: The aim of our study is to evaluate the feasibility and efficacy of the rendezvous technique for the treatment of iatrogenic ureteral injuries. **Methods**: From 2014 to 2019, 29 patients treated with the rendezvous technique for mono- or bilateral iatrogenic ureteral injuries were enrolled in this retrospective study. All the leaks were previously assessed by CT-urography and antegrade pyelography. Ureteral continuity was restored by performing the rendezvous technique, combining antegrade trans-nephrostomic access and a retrograde trans-cystostomic approach. A double J stent was antegradely inserted, and a nephrostomy tube was kept in place at the end of the procedure. A post-procedure CT-urography and a 30-day nephrostogram follow-up were performed. In the absence of a contrast leak, the nephrostomy tube was removed. Patient follow-up was set with CT-urography at 3, 6, and 12 months and stent substitution every 4 months. The CT-urography was performed to confirm the restored integrity of the ureter before stent removal. **Results**: The rendezvous technique was successful in all cases with the resolution of the ureteral leak. No major complications were observed. In all the patients, the nephrostomy tube was removed after 30 days. After performing CT-urography, the stent was removed permanently after 12 months. Only three cases showed local post-treatment stenosis treated with surgical ureteral reimplantation. **Conclusions**: The rendezvous technique is a safe and effective minimally invasive procedure that can be used to restore the continuity of the ureter, avoiding open surgery and providing valuable support for the management of complications after gynecological surgery.

## 1. Introduction

Ureteral trauma can be categorized into three possible etiologies: iatrogenic, blunt, and penetrating, which are responsible for 75%, 18%, and 9% of ureteral injuries, respectively [1,2]. The ureters are highly susceptible to iatrogenic injury due to their proximity to vital abdominal and pelvic organs, their close position to vascular structures, and their course along the retroperitoneum and upper pelvis, which makes them difficult to identify during surgical procedures [3,4].

For these reasons, ureter injury is the most common urological complication of pelvic surgery, with an incidence from 1% to 10% [5].

With the rapid expansion of minimally invasive laparoscopic procedures, the leading cause of iatrogenic ureteral injury has shifted from urologic to gynecologic surgeries: a reported 64% of iatrogenic ureteral injuries are related to laparoscopic gynecologic surgeries, while the remaining 26% and 11% occur during general surgical procedures and urologic procedures, respectively [2,4]. In particular, the ureteral injuries incidence ranges from 0.4% to 2.5% for laparoscopic hysterectomy [6] and is about 0.02% for vaginal hysterectomy [7]. From the urologist’s perspective, ureteroscopy is the most common cause of ureteral damage [2]. Colon and rectal open procedures, such as low anterior resection and abdominal perineal resection, are responsible for 9% of all cases of ureteral injury [5]. Other cases of iatrogenic ureteral injuries of the distal ureter are radiation therapy and appendectomy [8,9,10].

The distal third of the ureter is the most susceptible part to iatrogenic damage, accounting for 91% of injuries [4,11]. Iatrogenic ureteral injuries may be monolateral or bilateral, incomplete, or complete. In incomplete sections, a thin continuity between the proximal and distal stumps is recognizable in both computed tomography (CT)-urography and pyelogram images, while in complete sections, the opacification of the distal stump and the bladder cannot be demonstrated [12,13]. The American Association for the Surgery of Trauma (AAST) classifies ureteral injuries into five grades based on severity, ranging from minor contusions to complete avulsions with significant devascularization [14] (Table 1).

Ureteral injury may be recognized intraoperatively and treated without any complications. It may be overlooked and give symptoms in the immediate or late postoperative period, leading to increased morbidity and threatening kidney viability [15].

These injuries were historically repaired through standard open surgery: traditional methods included end-to-end uretero-ureteral anastomosis, ureteral reimplantation, ureteroneocystotomy, or some combination [11,15,16]. However, open surgery is accompanied by higher perioperative morbidity, prolonged hospitalization, and higher treatment costs [17].

Currently, percutaneous and endoscopic minimally invasive techniques represent alternative strategies for the management of ureteral iatrogenic injuries and have been proposed to reduce morbidity and the duration of hospitalization [17,18,19].

An integrated management approach involving radiologists and gynecologists can successfully be used to treat ureteral iatrogenic leaks through a minimally invasive radiological intervention, which is known as a “ureteral rendezvous technique” [8,20]. This technique combines an antegrade and a retrograde approach to increase the success rates of antegrade ureteral stenting in the more challenging cases of ureteral strictures or urinary leaks [21]. In the literature, successful results of this procedure have already been demonstrated [8,20,21].

Given the advantages and risks associated with minimally invasive gynecologic surgery, there is a critical need to develop effective techniques for managing iatrogenic ureteral injuries, which are among the most common urological complications of pelvic surgeries. The aim of this study is to report our experience with the rendezvous technique and evaluate its feasibility and effectiveness for re-establishing ureteral continuity in cases of iatrogenic ureteral injuries.

## 2. Materials and Methods

All the methods and procedures used were in accordance with the Declaration of Helsinki, and the institutional review board approved this study. All the patients treated with the rendezvous technique at our uro-radiological department in the period ranging from January 2014 to July 2019 were considered. The inclusion criteria were: monolateral or bilateral iatrogenic ureteral injuries, the presence of a complete or incomplete ureteral leak, injuries that occurred during gynecological, general, or urological surgical procedures; patients with non-iatrogenic ureteral injuries, comorbidities, and who had undergone chemotherapy or radiotherapy either before or after surgery were excluded.

Written informed consent was obtained from all the individual participants included in the study.

The diagnosis of ureteral transection injury was suspected based on the patient’s history and presence of symptoms in the early postoperative period (flank and loin pain, low-grade fever without any signs of septicemia, oliguria, or anuria) and subsequently confirmed by imaging.

**Figure 1:**
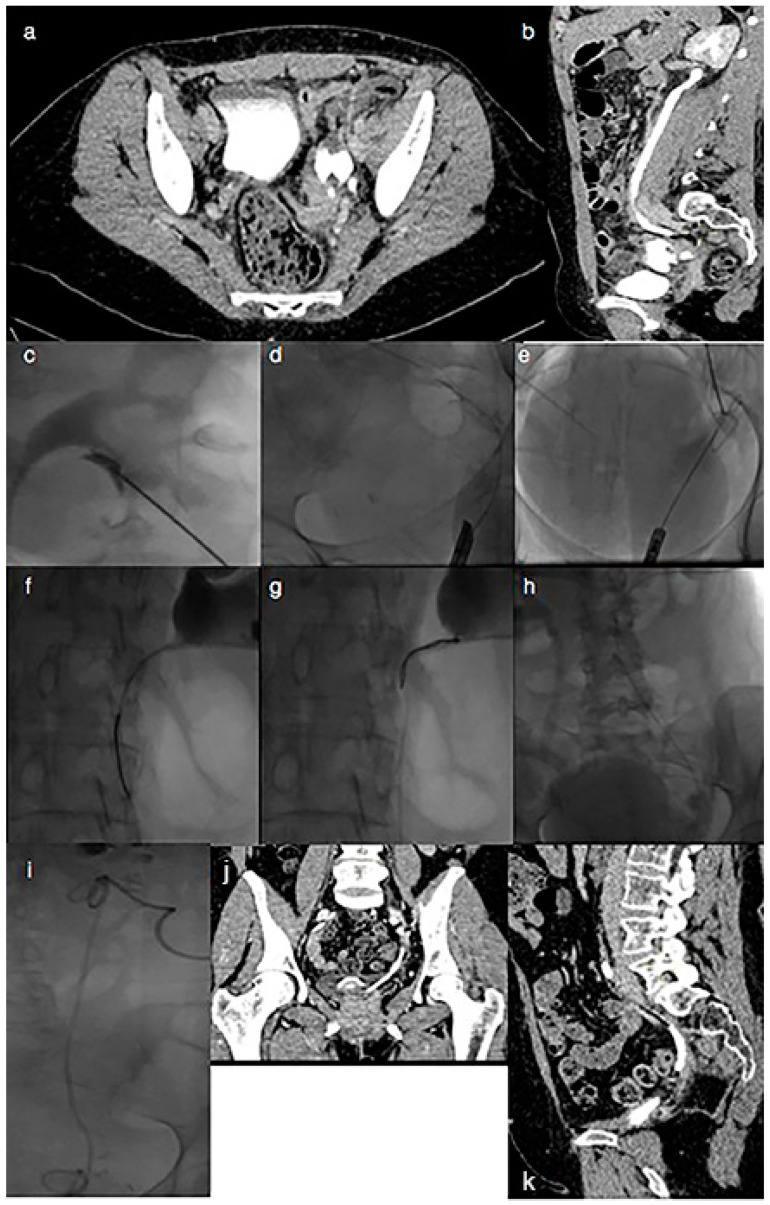
A 51-year-old woman with a history of malignant pelvic disease underwent abdominal radical hysterectomy. Abnormally increased levels of blood urea and creatinine were reported after 13 days from the surgical primary procedure. Axial, (**a**), and sagittal, (**b**), CT-urography images showed extravasation of contrast medium from the distal portion of the left ureter. The iatrogenic ureteral lesion was treated with the rendezvous technique, (**c**–**h**): using a flexible cystoscope, gynecologists inserted a guidewire in the distal stump; simultaneously, under fluoroscopic control and through nephrostomic access, interventional radiologists used a gooseneck snare to capture the end of the retrograde ureteric wire, which was retrieved from the percutaneous sheath. Then, an 8 French 24–26 cm double J stent was antegradely inserted by applying bidirectional traction on the guide, (**i**). Axial, (**j**), and sagittal, (**k**), CT-urography images performed at the time of stent removal, after about 6 months, confirmed the restored integrity of the ureter.

**Figure 2:**
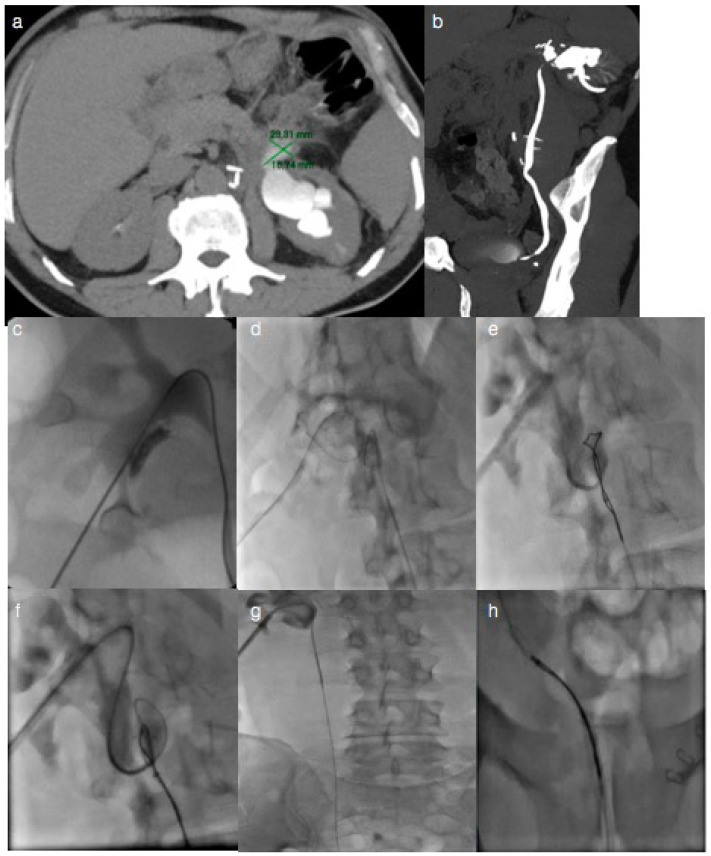
A 62-year-old woman presented increased blood urea and creatinine levels after retroperitoneal metastatic lymph node removal, located near the renal pelvis. Axial, (**a**), and sagittal, (**b**), CT-urography images showed extravasation of contrast medium from the proximal portion of the left ureter. The rendezvous technique was the treatment of choice, (**c**–**h**): from the nephrostomic access a guidewire, at first, and a 5Fr Berenstein catheter, subsequently, were placed in the extremity of the proximal stump; simultaneously, using a flexible cystoscope, urologists inserted a gooseneck catheter, emerging from the opposite site of the ureteral section, to capture the guidewire, which was pulled out and externalized from the urethra, realigning the stumps and allowing for placement of the double J stent. The integrity of the ureter was restored without any complications and the ureteral stent was then removed after 6 months.

All the patients underwent both a CT-urography and an antegrade pyelogram in order to diagnose the ureteric transection demonstrated by contrast medium leak from the ureter. Extravasation of contrast medium, without distal ureteral stump opacification, represented the hallmark sign of complete ureteral transection injury and indicated the site of the ureteral leak (Figure 1a,b and Figure 2a,b).

According to the AAST classification, ureteral injury was categorized into incomplete lesions, which include Grade II (laceration < 50% of the circumference) and Grade III (laceration > 50% of the circumference), and complete lesions, which are classified as Grades IV and V [14] (Table 1).

### Procedure Technique

A preoperative evaluation was obtained for each patient, including blood coagulability tests and a detailed history of any hypersensitivity to drugs or contrast media.

For all the patients, cystoscopy with retrograde ureteral stent placement and anterograde ureteral stent placement, individually, were considered among first-line treatment options. After the failure of these two therapeutic approaches due to the length of the defect and severe misalignment of the opposing ends of the ureter, we considered attempting the rendezvous technique as a combined access, trans-nephrostomic anterograde and transvescical retrograde approach to re-establish the ureteral continuity.

The initial management consisted of the placement, under local anesthesia, of a percutaneous 8-10F nephrostomy tube to alleviate the urine leak and prepare the percutaneous antegrade access for the rendezvous.

On the same day, or at least on the subsequent days, the rendezvous approach was performed by the interventional radiology team in conjunction with the uro-gynecological team capable of accomplishing cystoscopy in the angiography room.

During the procedure, the patient lies in a dorsal lithotomy position, with the flank homolateral to the elevated nephrostomy.

A deep sedation (Fentanyl 0.1 mg/^2^ mL and Propofol 5 mg/mL) was performed by the anesthesiologist according to the ventilation rate and level of consciousness.

All the patients received intravenous administration of prophylactic antibiotics (2 gr of intravenous Cefazolin).

Under fluoroscopic control, the IR take a hydrophilic 0.035-inch, 260 cm guidewire through the nephrostomy access to the level of the ureteral lesion.

Through retrograde catheterization of the ureteral ostium, previously performed using a cystoscope, using a hydrophilic guidewire, a gooseneck snare was inserted and used to capture, in the extra-luminal or intra-luminal space, the distal tip of the antegrade guidewire, which was pulled into the bladder lumen. Through a 4 French angiographic catheter, the hydrophilic guide was replaced with a Teflon-coated guide. Through a van Andel Peel-Away introducer, an 8 French 24–26 cm double J stent was antegradely inserted and successfully deployed to restore the alignment of the proximal and distal ends of the ureter, applying bidirectional traction on the guide (Figure 1c–h and Figure 2c–h).

At the end of the procedure, a nephrostomy tube was kept in place and left in situ for external drainage to prevent, in case of haematuria, an obstruction of the stent.

All the patients underwent a post-procedure contrast CT-urography and in the absence of complications were dismissed the following day with the nephrostomy tube opened until the next control.

The presence of hematuria, hemorrhage, or any signs of septicemia as a result of either the nephrostomy access itself or the subsequent intraureteral manipulations were considered major complications.

Follow-up consisted of a contrast antegrade nephrostomogram at 30 days, and, in the absence of a contrast leak, the nephrostomy tube was removed the same day as the nephrostomogram follow-up. The ureteral lesion was controlled with CT-urography at 3, 6, and 12 months.

The double J ureteral stent was kept in place to prevent ureteric strictures for a period of 12 months, with a retrograde stent substitution every 4 months to reduce urinary infections and stent obstructions.

All complications related to stent maintenance were assessed.

To assess renal function, blood urea and creatinine values were evaluated weekly for the first month and then monthly, considering normal ranges of 7–20 mg/dL and 0.5–1.2 mg/dL, respectively.

CT-urography was performed at the time of stent removal to confirm the restored integrity of the ureter.

Technical success was defined as the restoration of ureteral continuity by the accurate placement of a ureteral stent into the urinary tract.

Clinical success was defined as the absence of the need for re-external drainage or surgical reconstruction during the follow-up period.

## 3. Results

A total number of 29 patients, with complete and incomplete ureteral lesions, treated with the rendezvous technique, were evaluated. All the patients were females, with a mean age of 60 ± 15.4 years (range 42–88 years).

The etiology was related to gynecological surgery in 19 cases (65%), to rectal surgery in 6 cases (21%), and to urological surgery in 4 cases (14%).

The gynecological surgical procedures had been performed in all cases for malignant pelvic disease with radical laparotomic (LPT) hysterectomy. Six patients underwent LPT anterior rectal resection for rectal carcinoma, three patients underwent a laparoscopic (LPS) radical cystectomy for bladder carcinoma, and one patient underwent LPS removal of a retroperitoneal metastatic lymph node located near the renal pelvis.

In all these patients, abnormally increased levels of blood urea and creatinine arose in a median of 14 days (range: 5–32 days) after the surgical primary procedure. Fever and abdominal pain were reported and ureteral injury was suspected.

CT-urography and antegrade pyelography demonstrated evidence of a contrast medium leak from the ureter, confirming the ureteral lesion.

The time from the original surgery to the diagnosis of ureteral injury ranged from 8 days to 67 days (mean, 24.2 ± 23.2 days).

The iatrogenic ureteral lesions were localized in 17 (58.3%) cases in the left ureter and 12 (41.7%) in the right. In total, 25 lesions were unilateral (86%) and 4 were bilateral (14%). The anatomic location was the distal portion of the ureter in 27 (95%) cases and the proximal portion of the ureter in 2 patients (5%).

The ureteral injuries were incomplete (Grade II and III) in 75.9% of cases (n = 22; n = 2 Grade II; n = 20 Grade III) and complete (>Grade III) in 24.1% of cases (n = 7) [14].

In the complete injuries, the average extent of the lesion was 3.8 cm ± 1.2 cm, while in the incomplete injuries, the average extent was 2.3 cm ± 0.9 cm. All the patients reported the failure of traditional ureteral stenting performed using the conventional antegrade approach and were, subsequently, considered eligible for the rendezvous technique, which was performed using fluoroscopic antegrade and endoscopic retrograde ureteral access. The combined antegrade and retrograde ureteral approach for re-establishing ureteral continuity was successfully attempted in all 29 cases.

A technical success rate of 100% was achieved.

None of the procedures needed conversion to an open procedure. No intraoperative complications were reported.

The average operative time was 66.8 ± 20.6 min (range 35–92 min), with a median radiation time of 20 ± 6 min for monolateral and 44 ± 18 min for bilateral procedures.

The whole procedure was well-tolerated by all the patients and no major complications occurred.

After the procedure, contrast CT-urography demonstrated the absence of a ureteral leak and a well-positioned stent and nephrostomy in all the patients.

In all the patients, the nephrostomy tube was removed after 30 days in the absence of evidence of a ureteral leak through a contrast antegrade nephrostomogram.

The mean hospital stay, from the diagnosis time to the hospital discharge after the rendezvous technique, was 4.6 ± 2.3 days (range 2–12 days). No late ureteral leak was observed during the CT-urography follow-up.

The ureteral stent was permanently removed, in the absence of complications, after 12 months.

Minor complications, mainly dysuria and suprapubic discomfort, were observed in five patients. The blood urea and creatinine values gradually decreased and normal renal function was restored in all the patients during the first 4 weeks after intervention.

The CT-urography performed at the time of stent removal confirmed the restored integrity of the ureter in all the patients (Figure 1j,k).

No case of severe upper-tract infection requiring hospitalization, intravenous antibiotics, stent replacement, or open surgical manipulations was observed.

**Figure 3:**
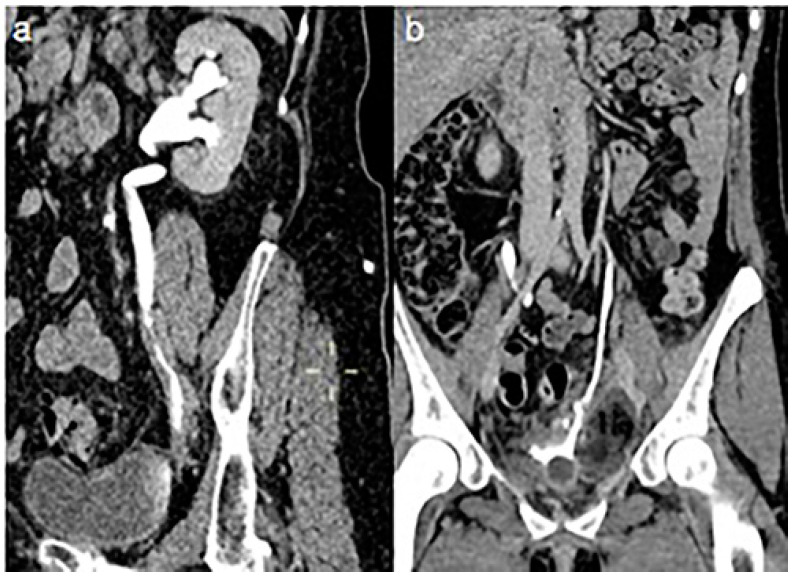
A sagittal, (**a**), CT-urography image of a 42-year-old woman demonstrated a post-treatment local stenosis after 45 days by stent removal in a patient with a lesion of the left distal ureter treated with the rendezvous technique 1 year before. This local complication was successfully treated with surgical ureteral reimplantation, (**b**).

In only three cases (10.3%), a post-treatment local stenosis was documented after 4 and 6 months from stent removal with symptom recurrence and, after a failed attempt of primary ureteroplasty, a surgical reconstruction, performed using ureteral reimplantation, was required (Figure 3). In the remaining 18 cases, there was no leakage and/or ureteric stricture at the site of the previous injury. A clinical success rate of 89.7% was achieved.

During the follow-up, after definitive ureteral recanalization and stent removal, all the patients reported no clinical abnormality. Normal serum urea (average: 14.71 mg/dL) and creatinine values (average: 0.85 mg/dL) were documented.

All the lesion characteristics distributions, procedural data, and complications are summarized in Table 2.

## 4. Discussion

The ureter is a vulnerable anatomic structure and can easily be compromised along its course during pelvic or abdominal surgery [20]. Furthermore, the advent of endoscopic surgery and the introduction of new complex ureteroscopic and laparoscopic techniques have been associated with a higher rate of iatrogenic ureteral trauma [2,21].

The incidence of ureteral lesions varies between 0.1% and 30% depending on the type of surgical intervention as well as the surgeon’s skill and experience. Only one-third of cases of iatrogenic lesions are detected during the procedure [8]. Intraoperative recognition and the subsequent immediate treatment of ureteral trauma are important, ensuring an excellent prognosis, [18] while a postoperative diagnosis leads to progressive deterioration of renal function. Furthermore, delayed diagnosis seems to be the single controllable factor adversely influencing the outcome [8,22]. In complex cases, the diagnosis of such lesions can benefit from artificial intelligence techniques [23].

Ureteric discontinuity following injury has been traditionally treated by open surgery, which is still the mainstream therapeutic approach, though a variety of new advanced surgical procedures can be employed. In particular, laparoscopic-assisted and robotic-assisted surgery is growing rapidly in urology and the reported results on the outcomes are equivalent to the open approach [4,24].

Interventional radiology offers minimally invasive treatment options for various pathological conditions through the use of increasingly advanced and safer devices [25,26].

In partial ureteral transections, cystoscopy with retrograde ureteral stent placement is considered among the first-line treatment options, but if it fails, an interventional radiologist may attempt anterograde ureteral stent placement [2,4]. In 2008, Ustunsoz et al. demonstrated, for the first time, the utility of interventional radiologic techniques in managing 22 patients with distal ureteral injuries diagnosed late after caesarean section without any major complications associated with percutaneous management [27].

Reports from the English literature described endoscopic realignment of ureteric injuries with the rendezvous technique using a flexible antegrade scope and a rigid retrograde ureteric scope [8,24]. Liu et al. described a technique for endoscopic realignment of completely transected ureters with combined antegrade and retrograde ureteroscopy [28], and Pastore et al. reported a small case series of 18 patients treated using this procedure, with an overall success rate of 78% [8].

Chang et al. also described, in their case report, a patient who developed delayed urine leakage following partial sacrectomy for recurrent mucinous appendiceal malignancy, which was managed using the rendezvous technique [29].

Our results suggest that the rendezvous technique allowed the restoration of ureteral integrity in the case of iatrogenic ureteral transection injury and is both feasible and highly effective. The technique yielded encouraging results with a short hospital stay (mean 4.6 days), no perioperative complications, and avoidance of the need for an open surgical procedure with a long-term solution. Minor complications, mainly dysuria and suprapubic discomfort, were attributed to bladder irritation by the stent. The stents were well-tolerated by the rest of the subjects without any restriction on everyday activities.

All the patients had a failed initial attempt at inserting the ureteral stent, either antegradely by percutaneous nephrostomy or retrogradely by cystoscopy. Subsequently, ureteroscopic manipulation combined with antegrade radiological techniques was used as a significant alternative to invasive surgery.

In accordance with the data reported by Arabi et al. [30], in our cases, we used some of the identified technical teaching points, allowing the fluoroscopy-guided realignment of total ureteric transection. First, the presence of a collection cavity due to a urine leak facilitated capturing the retrograde ureteric wire, even though in many cases, the initial placement of a diversion nephrostomy may have led to the complete obliteration of the cavity and subsequently precluded snaring maneuvers. Second, the antegrade sheath was advanced into the collection or beyond the edge of the transected ureter to prevent de-gloving of the ureter during wire retrieval from the retrograde sheath. In contrast to what was reported by Arabi et al. [30], in our study, we introduced the tapered end of the double J stent antegradely into the trans-nephrostomic sheath and there was no case of intussusception of the distal portion of the pelvic ureter into the urinary bladder.

A recent study by Fontana et al. reported the use of minimally invasive techniques for managing ureteral iatrogenic leakage after gynecologic surgery. The study included 15 patients, with a technical success rate of 80% using antegrade and retrograde approaches (rendezvous technique) in 3 cases after the failure of the antegrade technique. Our study aims to evaluate the rendezvous technique alone in 29 patients with complete and incomplete ureteral transection injuries, demonstrating a 100% technical success rate [31].

However, while this technique is a relatively non-invasive procedure compared with major surgical repair and may obviate open surgical repair, it has potential complications. Ureteric intussusception during the antegrade introduction of a double J stent was described in a previous case reported by Pastore et al. [8], which was managed by introducing the stent retrogradely. In our study, we did not report any case of ureteric intussusception.

In 2020, in a study conducted on 19 patients with ureteral injuries (leaks, strictures, or both), Kawada et al. reported a technical success rate of 89.5% and a clinical success rate of 68.4%, with only 4 patients (21.2%-of which two leakages with strictures and two strictures) achieving a stent-free condition. In their study, the rendezvous technique was used for both ureteral leaks and strictures, employing a self-made snare inserted via the PN tract to capture the guidewire in the ureter or retroperitoneal space [32].

In our study, which specifically focused on treating post-surgical ureteral leaks, we achieved a technical success rate of 100% using a commercially available gooseneck catheter, retrogradely inserted for better maneuverability to capture the distal tip of the antegrade guidewire. Moreover, the rendezvous technique was performed in the extra-luminal space for complete leaks (3) due to the larger extent of the injury and in the intra-luminal space for incomplete leaks, allowing for the capture of the guidewire inside, resulting in less traction on the ureter.

Regarding correct stent positioning, it is always considered necessary to ensure ureteral integrity in the post-intervention period [33,34]. The reasons underlying stent use are to achieve ureteral healing, prevent urinary extravasation, and avoid or reduce the possibility of stricture relapse developing [34]. The optimal stent size and removal time remain controversial. In the present study, we consistently used the same hydrophilic double-J stent with a diameter of 8F for all the patients, and the stents were then removed after a duration of 12 months. Follow-up CT-urography after stent removal confirmed the restored integrity of the ureter in all the patients. Strictures at the site of ureteric injury after stent removal are commonly encountered and reported in the literature in a range between 12 and 30% of the treated population and can be managed by ureteroplasty and/or surgical reconstruction [33,34]. In our series, subsequently, only three cases (10.3%), exhibiting local post-treatment stenosis related to complete ureteral injuries (>Grade III), received surgical ureteral reimplantation due to ureteroplasty failure after stent removal.

Our study is the only one in the literature to have considered the largest number of patients treated with the rendezvous technique for ureteral leaks. However, the main limitations of this study are the small number of patients, reflecting the rarity of this iatrogenic injury, and its retrospective nature. It will be unlikely that a larger group of patients can be enrolled and that a randomized clinical trial comparing the various minimally invasive or major surgical approaches can be performed.

## 5. Conclusions

In conclusion, our study demonstrates that percutaneous rendezvous realignment is a viable first-line treatment option for patients with iatrogenic ureteral injuries due to incomplete transection, which are quite common after pelvic surgery. This technique should also be considered as an alternative for complete injuries, although there is a higher likelihood of subsequent stenosis. This technique offers feasibility, efficacy, and a low rate of peri- and post-procedural complications, making it advantageous in the management of such cases, with the experience of the involved radiologists in choosing the appropriate operating approach being crucial for optimizing the outcomes.

## Figures and Tables

**Table 1:** AAST ureteral trauma grading score [14].

Grade	Injury
I	Periureteral hematoma
II	Laceration < 50% of the circumference
III	Laceration > 50% of the circumference
IV	Complete tear < 2 cm revascularized
V	Complete tear < 2 cm revascularized

**Table 2:** Lesion characteristics distributions, procedural data, and complications.

Charactesistics	Values
median age (mean and standard deviation)	60.0 ± 15.4
***sex (n; %)***	
female	29
males	0
***etiology (n; %)***	
gynecological surgery	19 (65)
rectal surgery	6 (21)
urological surgery	4 (14)
***diagnosis (n; %)***	
CT-urography	29 (100)
antegrade pyelography	29 (100)
mean time from original surgery to diagnosis (mean and standard deviation)	24.2 ± 23.2
***lesion location (n; %)***	
left ureter	17 (58.3)
right ureter	12 (41.7)
***lesion type (n; %)***	
unilateral	25 (86)
bilateral	4 (14)
***lesion anatomic location (n; %)***	
distal ureter	27 (95)
proximal ureter	2 (5)
mean operative time (mean and standard deviation)	66.8 ± 20.6
mean hospital stay (days; mean and standard deviation)	4.6 ± 2.3
***post-procedural complication (n; %)***	
major	0 (0)
minor	5 (17)
stenosis after stent removal (n; %)	3 (10.3)
ureteral reimplantation (n; %)	3 (10.3)

## Data Availability

No new data were created or analyzed in this study. Data sharing is not applicable to this article.
